# Supplementary material for: The feasibility of the posterior tibial nerve-flexor hallucis brevis pathway applied in neuromuscular monitoring: a multicentric, controlled, and prospective clinical trial
Source: PeerJ. 2024 Mar 26;12:e17154. doi: 10.7717/peerj.17154 (PMC10979752; doi:10.7717/peerj.17154)
Supplement: Supplemental Information 1 [file peerj-12-17154-s001.zip › Raw data/table 2/Table 2.docx]

**Table 2 Comparisons between thumb and toe monitoring results**

|  | Mean±SD | | Difference (hand minus foot) |  |  |
| --- | --- | --- | --- | --- | --- |
| Period | At hand | At foot | Mean±SE (95% CI) | t | P |
| OT (s) | 232.01±65.30 | 285.55±71.87 | -53.54±4.66 (-62.76 to -44.33) | -11.48 | <0.001 |
| NTR (min) | 56.21±9.56 | 58.70±9.92 | -2.49±0.49 (-3.45 to -1.53) | -5.12 | <0.001 |
| SRT (min) | 14.93±5.54 | 11.72±5.38 | 3.22±0.58 (2.07 to 4.37) | 5.53 | <0.001 |
| TT (min) | 94.91±14.60 | 89.02±14.84 | 5.89±0.81 (4.28 to 7.49) | 7.27 | <0.001 |

Student’s paired t-test was used and the 95% CI of mean difference was calculated using standard error.

SE, standard error.
