# Supplementary material for: The feasibility of the posterior tibial nerve-flexor hallucis brevis pathway applied in neuromuscular monitoring: a multicentric, controlled, and prospective clinical trial
Source: PeerJ. 2024 Mar 26;12:e17154. doi: 10.7717/peerj.17154 (PMC10979752; doi:10.7717/peerj.17154)
Supplement: Supplemental Information 1 [file peerj-12-17154-s001.zip › Raw data/table 3/Table 3.docx]

**Table 3 The difference between hand and foot and its comparisons among three centers**

|  | Difference (hand minus foot) | | | ANOVA | | P-value of post-hoc comparisons | | |
| --- | --- | --- | --- | --- | --- | --- | --- | --- |
| Period | Center S | Center G | Center J | F | P | S vs. G | S vs. J | G vs. J |
| OT (s) | -43.15±52.25 | -63.94±63.13 | -64.68±60.61 | 2.64 | 0.075 | 0.078 | 0.052 | 0.955 |
| NTR (min) | -1.70±6.01 | -4.04±6.23 | -2.67±5.76 | 1.86 | 0.159 | 0.057 | 0.404 | 0.322 |
| SRT (min) | 3.33±7.20 | 2.38±6.73 | 3.83±5.33 | 0.39 | 0.678 | 0.508 | 0.732 | 0.392 |
| TT (min) | 6.52±10.26 | 6.51±8.27 | 4.07±5.82 | 0.88 | 0.419 | 0.993 | 0.211 | 0.299 |

One-way ANOVA and Fisher’s LSD test as post-hoc comparison was used.
