# Supplementary material for: The feasibility of the posterior tibial nerve-flexor hallucis brevis pathway applied in neuromuscular monitoring: a multicentric, controlled, and prospective clinical trial
Source: PeerJ. 2024 Mar 26;12:e17154. doi: 10.7717/peerj.17154 (PMC10979752; doi:10.7717/peerj.17154)
Supplement: Supplemental Information 1 [file peerj-12-17154-s001.zip › Raw data/table 4/Table 4.docx]

**Table 4 Two-way mixed-design ANOVA results of monitoring results between thumb and toe among three centers**

|  | Site (hand/foot) | | Center (S/G/J) | | Interaction (Site x Center) | |
| --- | --- | --- | --- | --- | --- | --- |
| Period | F | P | F | P | F | P |
| OT (s) | 135.96 | <0.001 | 0.55 | 0.577 | 2.64 | 0.075 |
| NTR (min) | 29.89 | <0.001 | 5.26 | 0.006 | 1.86 | 0.159 |
| SRT (min) | 26.02 | <0.001 | 1.58 | 0.211 | 0.39 | 0.678 |
| TT (min) | 43.46 | <0.001 | 6.82 | 0.002 | 0.88 | 0.419 |

Two main effects (site and center) and one interaction effect (site x center) were reported with F-value and P-value.
