# Supplementary material for: The feasibility of the posterior tibial nerve-flexor hallucis brevis pathway applied in neuromuscular monitoring: a multicentric, controlled, and prospective clinical trial
Source: PeerJ. 2024 Mar 26;12:e17154. doi: 10.7717/peerj.17154 (PMC10979752; doi:10.7717/peerj.17154)
Supplement: Supplemental Information 1 [file peerj-12-17154-s001.zip › Raw data/table 5/Table 5.docx]

**Table 5 Correlation coefficient analysis between monitoring results and demographics**

|  | Pearson's r | | | |  | Spearman's rho | | |
| --- | --- | --- | --- | --- | --- | --- | --- | --- |
| Period | Age, year | Height, cm | Weight, kg | BMI, kg/m2 |  | Gender | ASA grade | Surgical type |
| OT (s) | 0.078 | 0.148 | 0.139 | 0.092 |  | -0.045 | 0.384 | 0.014 |
| NTR (min) | -0.145 | -0.012 | -0.102 | -0.101 |  | 0.100 | 0.205 | 0.150 |
| SRT (min) | 0.105 | 0.089 | 0.086 | 0.051 |  | 0.018 | 0.102 | 0.022 |
| TT (min) | 0.051 | 0.017 | 0.031 | 0.043 |  | -0.026 | 0.605 | 0.086 |

Pearson’s correlation coefficient was used between two continuous variables while Spearman’s rho was used if one or both variables were categorical.

No significant correlation coefficient (P<0.05) was found in these results.
