# Supplementary figures and images for: The feasibility of the posterior tibial nerve-flexor hallucis brevis pathway applied in neuromuscular monitoring: a multicentric, controlled, and prospective clinical trial
Source: PeerJ. 2024 Mar 26;12:e17154. doi: 10.7717/peerj.17154 (PMC10979752; doi:10.7717/peerj.17154)

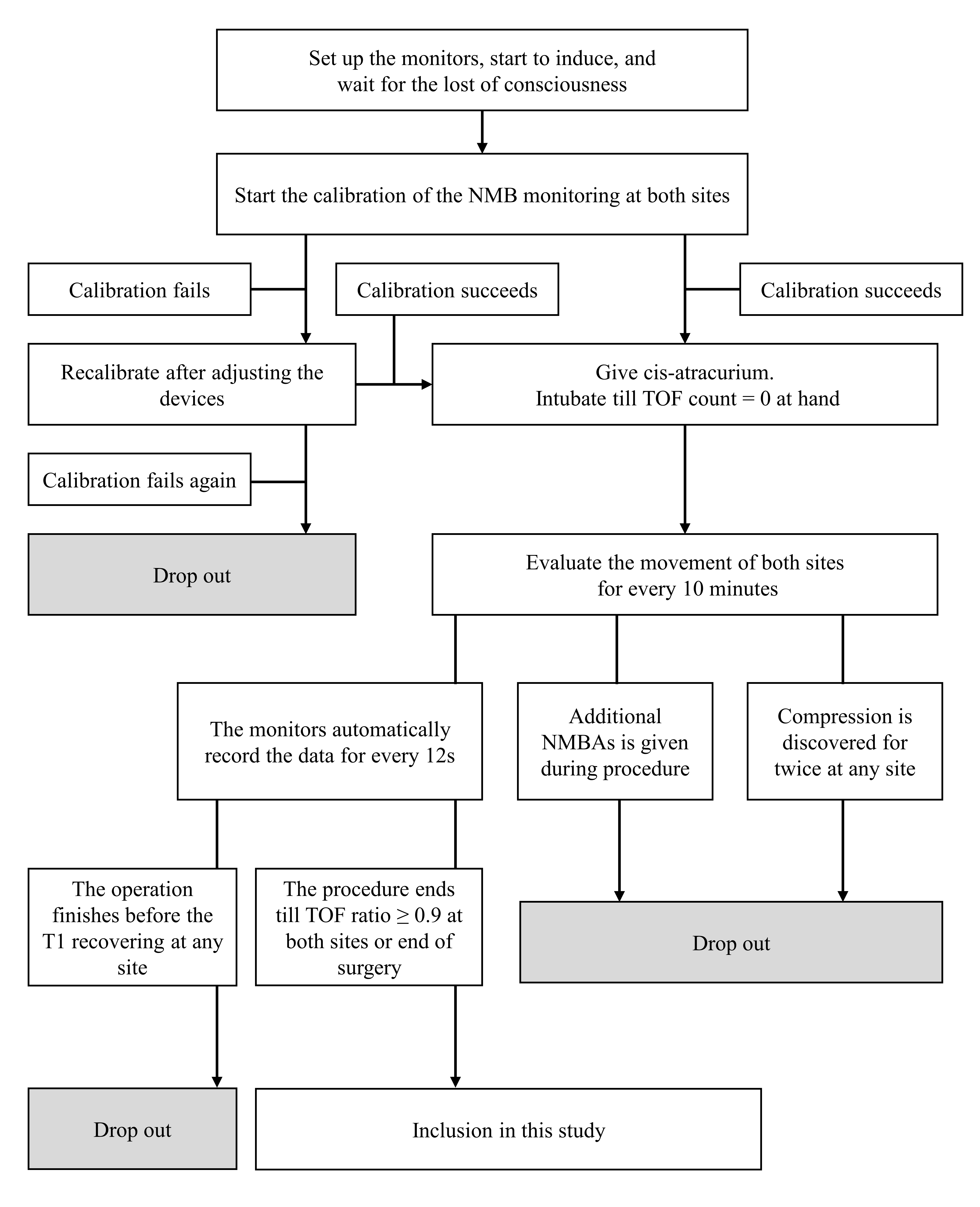

Supplement: Supplemental Information 1 [file peerj-12-17154-s001.zip › Raw data/Figure 1/Figure 1.tif]

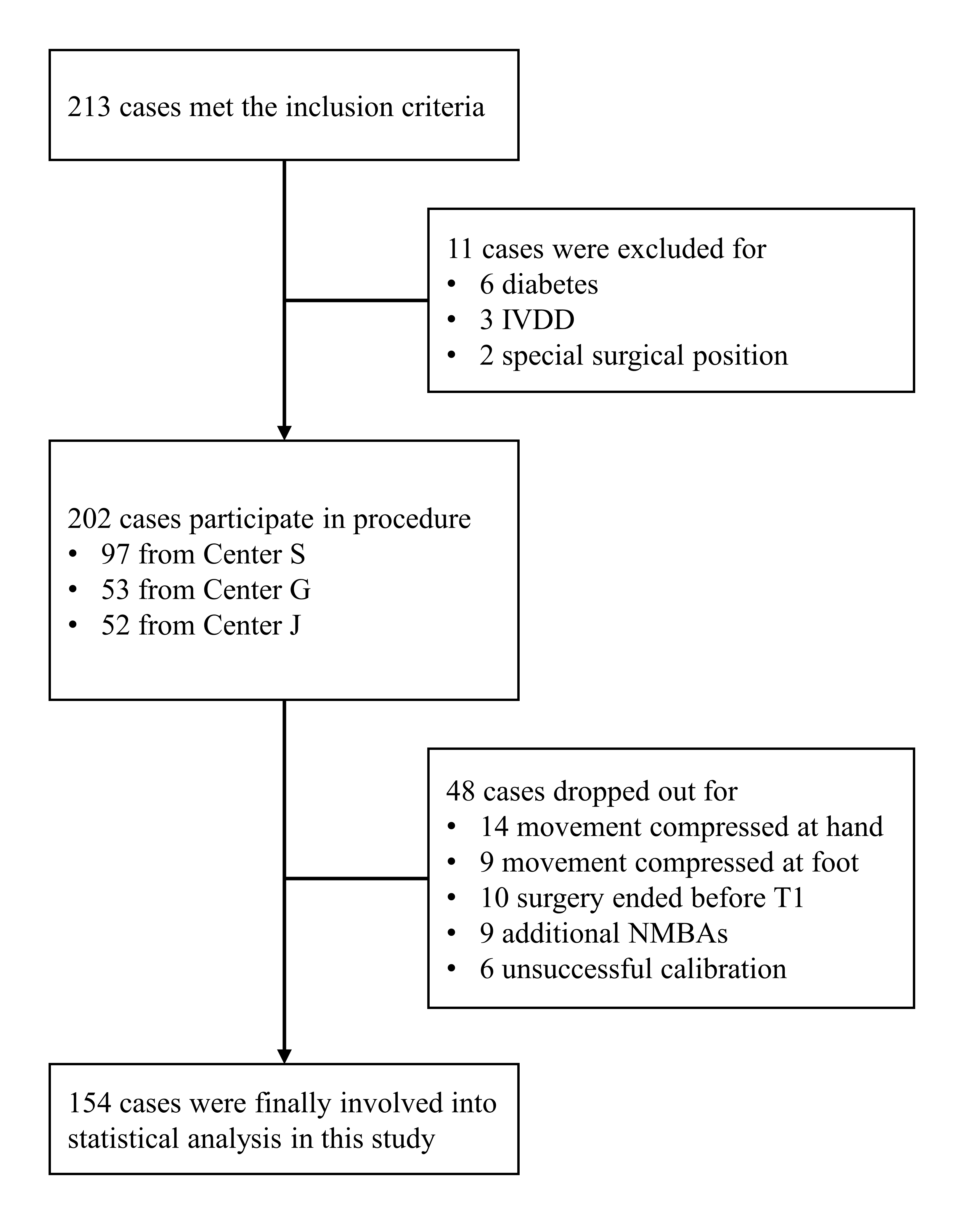

Supplement: Supplemental Information 1 [file peerj-12-17154-s001.zip › Raw data/Figure 2/Figure 2.tif]
