# Supplementary material for: The feasibility of the posterior tibial nerve-flexor hallucis brevis pathway applied in neuromuscular monitoring: a multicentric, controlled, and prospective clinical trial
Source: PeerJ. 2024 Mar 26;12:e17154. doi: 10.7717/peerj.17154 (PMC10979752; doi:10.7717/peerj.17154)
Supplement: Supplemental Information 3 [file peerj-12-17154-s003.docx]

**Clinical Trial**

**The Feasibility of the Posterior Tibial Nerve-****Flexor Hallucis Brevis Pathway Applied in Neuromuscular Monitoring: A Multicentric, Controlled, and Prospective Clinical Trial**

Catalogue

1. research background
2. Clinical data and methods

2.1 Clinical data

2.1.1 Study Purpose

2.1.2 Test design

2.1.3 Case selection

2.2 Research Ethics and Registration

2.3 Sample size calculation

2.4 Test equipment

2.5 Test methods and steps

2.5.1 Screening

2.5.2 Anesthesia procedure

2.5.3 Data records

2.6 Observed indicators

2.7 Data acquisition method

2.8 Data quality assurance method

2.9 Data and Statistical Methods

three.matters need attention

four.reference documentation

## research background

In modern medicine, muscle relaxants have been widely used in clinical anesthesia and respiratory support and respiratory therapy in critically ill patients. Because different individuals for muscle relaxant sensitivity and responsiveness is very different, combined with the effect of muscle relaxants by volatile anesthetics, intravenous anesthetics, aminoglycan antibiotics and the patient's age, body temperature and other factors, so by monitoring the application of muscle muscle transmission function block and recovery status, to reduce the incidence of various serious complications caused by residual muscle relaxation, improve the safety and rationality of clinical application is necessary [2].

The emergence of muscle relaxation monitor, this research opened up a wider space. At present, the application of muscle relaxation in surgical anesthesia monitor has been widely recognized, but not popular to clinical anesthesia work, most clinical anesthesia or with clinical experience evaluation judgment postoperative patients extubation time, for postoperative muscle loose residual, patients with delayed respiratory depression, awakening delay, hypoxemia [3] occurs, and this is postoperative respiratory complications and even respiratory depression risk is the most common cause of [4]. MurphyGS et al found that the use of a muscle relaxation monitor reduced the incidence of residual retention of ventricular relaxation and the recovery of respiratory complications [5]. In 2010, Murphy et al [6] summarized 15 clinical studies from 2000 to 2008 and found the incidence of residual muscle ranging from 3.5% to 88%. In recent years, more and more studies have proved that the implementation of intraoperative muscle relaxation monitoring (especially quantitative monitoring) can guide clinicians to use muscle relaxation drugs more rationally, and help to reduce the occurrence of residual muscle relaxation after surgery. Bailard et al [7] studied the occurrence of residual muscle relaxation in 1995,2000,2000, and 2004 in a French hospital and found that the incidence of postoperative residual muscle relaxation decreased significantly with the increased use of muscle relaxation monitoring and muscle relaxation antagonism. Therefore, the incidence of residual muscle relaxation is closely related to the strictness of the diagnostic criteria, and under other conditions unchanged, strengthening muscle relaxation monitoring and improving the diagnostic criteria will reduce the incidence of residual muscle relaxation.

At present, common methods for monitoring muscle relaxation include mechanical kinogram (Mechanomyography, MMG), electromyography (Electromyography, EMG), and acceleration kinogram (Acceleromyography, AMG) [8]. Mechanical kinogram is the standard method to monitor muscle relaxation by detecting the actual muscle strength of the muscle. However, this kind of man-machine connection of the monitor has many restrictions, which is not easy to operate, and is sensitive to the displacement, and the monitoring results are obviously disturbed [9]. EMG monitors the composite action potentials caused by the stimulation of a single motor nerve, but its consistency with mechanical EMG is not high, and it is susceptible to pseudodifference, which cannot be used as an effective alternative method [10]. The acceleration kinogram method is to monitor the muscle relaxation [11] by stimulating the peripheral motor nerves with electricity to reach a certain stimulation intensity (threshold) and monitoring the movement amplitude produced by the controlled muscle contraction. The single root muscle fibers in response to stimulation follow an all-or-none pattern, while the overall muscle strength of the muscle population depends on the number of muscle fibers involved in the contraction. If the stimulus intensity exceeds the threshold, all the innervated muscle fibers contract, and the muscle produces the maximum contractile force. After the administration of muscle relaxation drug, the degree of reduced muscle reactivity is in parallel with the number of blocked muscle fibers. Keeping the degree of super stimulation unchanged, the measured strength of muscle contractility can indicate the degree of neuromuscular blockade.

1994 The Accelerator muscle relaxation monitor has been developed to provide a reliable method for assessing neuromuscular blockade by stimulating the ulnar nerve and monitoring its TOF values in the adductor pollicis muscle of the wrist. As the study unfolds, muscle relax monitoring has proven to be used to guide key procedures in clinical anesthesia.[12] When the TOFcnt is 0, observing endotracheal intubation, accurately grasping the intubation time; in [13], when the muscle relaxation is detected at T1=25%, improving the satisfaction of anesthesia and reducing the use of muscle relaxation; when the TOF value is 25%, the additional muscle relaxation antagonist effectively reduces the resuscitation time of patients and postoperative muscle weakness [14]; [15] detects the residual condition of postoperative muscle relaxation through the head 5s and tongue plate test, providing valuable advice for the timing of muscle relaxation. Therefore,] = 1. Determine the timing of endotracheal intubation and extubation; 2. Maintain the appropriate muscle relaxation, meet the surgical requirements, and ensure the smooth operation of the operation; 3. Guide the use of muscle relaxant and the time of additional muscle relaxant; 4. Avoid phase arrest caused by excessive dosage of succinylcholine; 5. save the timing of muscle relaxation reversal and the dose of antagonistic drugs; 7. prevent postoperative respiratory insufficiency caused by the residual effect of muscle relaxant.

Now, the stimulation of the ulnar nerve to monitor the response of adductor hallicis (Adductorpollicis, AP) has become the gold standard [16] for clinical muscle relaxation monitoring, while the adductor, diaphragm, frown, orbicularis oculi muscle are the alternative options for muscle relaxation monitoring. However, usually because the surgical site involves these muscles or surgical position, muscle relaxation monitoring of laryngeal adductor and diaphragm can not be widely used in clinical use. During surgery, when the patient is operated by the head, face or arm, an electrical neurostimulator is usually applied to the leg to detect the degree of neuromuscular blockade after the application of non-depolarized muscle, and the foot as muscle relaxation monitoring is another option. While the flexor pollicis is the main muscle group in response to tibial nerve stimulation, so the big toe is considered the best position to measure the feet.

HEIER compared the difference of [17] with the gold standard thumb and found that the thumb TOF value recovered slower than the big toe. Given that the ulnar nerve consists of approximately 80% type I fibers and 20% type II fibers, type I fibers are much less represented in the composition of the posterior tibial nerve, and type I fibers were shown to be more sensitive to non-depolarized muscle relaxants compared to type II. Therefore, when the detection position is the finger, the deep anesthesia period and the complete recovery time should be longer than the toes. They proposed that clinically foot monitoring could allow faster monitoring of muscle relaxation recovery to guide additional muscle relaxants. Similarly, the TOSHIMITSU study also showed that monitoring the big toe with acceleration sensors would reach T1=25% faster than in the hand, potentially leading to an underestimation of neuromuscular blockade. If so, residual paralysis [9] may be present in the airway muscles when the neuromuscular function is assessed with the movement of the big toe. Therefore, the extent of neuromuscular blockade in the great hallux observed by using an electrical neurostimulator, by monitoring the maintenance dosage during surgery and for the evaluation of muscle relaxation effect is temporarily not exact. While many other studies [14,18-21] also compared the posterior tibial nerve with the ulnar nerve stimulation and found a faster recovery of the big toe than the TOF of the hand, monitoring the big toe may lead to a relative underestimation of neuromuscular blockade. So when the arm surgery is almost over, it is still recommended to move the monitor to the adductor hallicus for further monitoring. The above study only compared the difference between TOFR values detected at 75%, and to avoid postoperative residue, TOFR values have been recommended to recover to 90% before extubation [21]. Yasuyuki et al found that there was no clinically significant difference in monitoring muscle relaxation recovery in the big toe and thumb at recovery to TOFR90% [22]. Therefore, monitoring the relaxation of the big toe may cause neuromuscular blockade.

Through the above, we can find that stimulate the tibial nerve, monitoring the big toe neuromuscular recovery can be used as one of the NMT monitoring site selection [21], but compared with the ulnar nerve gold standard, the data failed to prove that it can completely replace the ulnar nerve status, considering the study patients number is less, and most of the single center, a single surgery comparison, for all is European and American race, so the test conclusion to bring certain limitations. Moreover, the literature listed in the above elaboration does not fully discuss the differences in intubation time, additional muscle relacant time, extubation time and postoperative residue during muscle relaxation monitoring, and still lacks key evidence on whether the foot can be used as an alternative choice for NMT monitoring site. Foreign compared the influence of muscle block drugs on the thumb contraction and big toe flexion, but often get controversial results, and the foot nerve monitoring results and the gold standard, this study hopes that through the clinical validation test based on the ulnar nerve gold standard, NMT monitoring the foot nerve muscular transmission TOFR foot equivalent interval, which can make the foot NMT in the future clinical application to provide theoretical basis.

**2.Clinical data and methods**

# 2.1 Clinical data

# 2.1.1 Test Purpose

Using ulnar nerve monitoring as the gold standard, the TOF values of the posterior tibial nerve and the ulnar nerve in the NMT monitoring, and the correlation between TOFR was calculated, to explore and verify the equivalent interval of the posterior tibial nerve versus the hand in the NMT monitoring.

# 2.1.2 Test design

This study uses a prospective, multicenter, open, controlled clinical trial design.

**prospect**

The posterior tibial nerve in neuromuscular transmission can be used as an alternative to the site, but the results of foot nerve monitoring still lag behind the gold standard, and there is no large sample multicenter clinical study.

**multicenter**

There are three research centers participating in the clinical trial, namely the Third Affiliated Hospital of Sun Yat-sen University, Jiangmen Central Hospital and the Second Affiliated Hospital of Guangzhou Medical University (among which the Third Affiliated Hospital of Sun Yat-sen University is the lead hospital).

**Method of enrollment**

This project is jointly undertaken by the three research centers, the Third Affiliated Hospital of Sun Yat-sen University, Jiangmen Central Hospital and the Second Affiliated Hospital of Guangzhou Medical University. This study will have at least 50 subjects and at most 100 subjects enrolled in each research center.

**2.1.3 Case selection**

Patients undergoing general anesthesia from Mar-2019 to Jan-2020 were selected and informed consent was signed. Screening according to the entry criteria: subjects who meet the enrollment criteria and do not meet the exclusion criteria were included in the trial process.

**target population**

Determine the target population: patients requiring general anesthesia and enter the screening period.

**Inclusion criteria**

1. aged 18-60 years;
2. graded as American Society of Anesthesiologists (ASA) Ⅰ or Ⅱ;
3. with a Body Mass Index (BMI) between 18-30kg/m^2^;
4. without any history or family history of diseases related to neuromuscular junction.

**Exclusion criteria**

1. history of nerve injury or/and use of drug affecting neuromuscular transmission;
2. dermatitis, ulcer or/and other lesion at the monitored sites;
3. history of diabetes, stroke, cerebral hemorrhage or/and intervertebral disc disorder;
4. severe cardiac, pulmonary, hepatic, renal dysfunction;
5. undergoing microsurgery or surgery requiring special body position.

**Withit (shedding) case criteria**

1. patient requests to withdraw consent;
2. the initial calibration fails twice or more;
3. any monitored site is discovered to be compressed twice or more;
4. any addition of NMBAs before the end of data collection;
5. T1 (TOFC = 1) did not recover before the end of surgery;
6. any severe adverse event happens during the monitoring.

**2.2 Research Ethics and Registration**

This study has been approved by the Ethics Committee of the Third Affiliated Hospital of Sun Yat-sen University ([2018] 02-348-01) and registered through the Chinese Clinical Trial Registry (in registration). In the process of the study, introduce the purpose, method and significance of the study to the patients or their family members in strict accordance with the ethical principles, ensure the informed consent of the patients or their family members, and sign the informed consent form.

**2.3 Sample size calculation**

According to the report by Heier and Hetland,[16] over 95% of the difference of onset time between the thumb and the toe fell within -21~-113 seconds, while the difference of spontaneous recovery time fell within -7~-1 minutes. Expecting for 99% of the difference in this study lying on the same section, with significance level (α) of 0.01, power of test (1-β) of 90%, drop-out rate of 20%, the sample size was calculated as 200 patients based on single-arm objective performance criteria.

## 2.4 Test equipment

## Drugs: midazolam, fentanyl, propofol, cis-atracuronium, remifentanil

Devices and instruments: Surgical Anesthesia System (Madston), Benevision N12 Patient Monitor (MNT)

## 2.5 Test methods and steps

**2.5.1 Screening**

## Before surgery, the investigator selected the patients according to the operation arrangement of the operation day, and signed the informed consent form. For those with full civil capacity, the informed consent shall be signed by the patient in person, and for those without civil capacity or limited civil capacity, it shall be signed by the patient's legal guardian. After signing, the preoperative screening form was filled in according to the medical records and the inclusion and exclusion criteria, and the screening number was given according to the order of the study center.

**2.5.2 Anesthesia procedure**

1) Preparation before induction:

All patients were admitted to the operating room by primary anesthesia. None of the patients had preoperative medication. Non-invasive blood pressure, ECG monitoring (ECG) and pulse oxygen saturation (SpO 2), and muscle relaxation monitoring (N M T). The mask received oxygen for 5 L/min, open upper limb venous access, intravenous 10 mL / kg (completed within the first hour).

2) Induction process and maintenance:

Anesthesia was induced by intravenous administration of midazolam 0.05-0.1mg/kg, propofol 2-2.5mg/kg, fentanyl 2-4 ug / kg. Until the patient lost consciousness, the patient was given 0.2mg / kg (4 x ED95). When the hand TOFcnt was 0, the anesthesiologist assessed the satisfaction of the subject's throat muscle relaxation, and performed endotracheal intubation after the muscle relaxation was satisfied. During the operation, after intubation, mechanical ventilation was performed by anesthesia machine. The ventilation mode was selected as volume-controlled ventilation mode, with tidal volume of 8-10 mL/kg, respiratory rate of 12 bpm, and breath ratio of 1:2. intraoperative, PetCO2 was maintained at 35-45mmHg, propofol and remifentanil, and the intravenous target control delivery mode was given according to the pharmacokinetic parameters. The initial plasma target concentration of propofol was set at 4-6 ug / ml and the target concentration of remifentanyl at 4-8 ng / ml, adjusted for the cycle fluctuation, each at ± 0.5 ug / ml (propofol) or 0.5 ng/ml (remifentanyl).

1. monitor

Install electrodes and sensors to fix the tested limb: the tested arm is straight and fixed on the arm bracket of the operating bed, the thumb is freely extended, and the other four fingers are fixed on the arm support with a strap. The lateral calf is straight, and the calf near the ankle is fixed to the operating bed. The insulation device was used to heat the subject, monitor the surface temperature of the palms and soles of the subject, and keep the body surface temperature above 32.5℃. Connect the corresponding accessories.

[1] Start period: When giving muscle relaxant, immediately start the muscle relaxation measurement of the hand and foot at the same time, and start the calibration of the tested equipment, obtain the super current and reference amplitude, and select the TOF measurement mode (2Hz, 12s interval) until the TOFcnt measurement value of hand and foot is 0. The anesthesiologist performed endotracheal intubation after testing the satisfaction with throat relaxation. The NMT measurement process avoids the touch of the subject's tested hands and feet.

[2] Failure period: Due to no additional muscle relaxant during the operation, the TOF interval was maintained at 12s interval, and the muscle relaxation monitoring continued. When the efficacy gradually subsided, record the TOF recovery to the corresponding TOF value of hand monitoring. The TOFR of the hands and feet reached 90% of the time and the trial ended.

After the completion of the clinical study, the subsequent surgery and anesthesia can be performed as usual according to the specific situation.

**2.5.3 Data records**

## 1) General situation

## Age, height, weight, past medical history, and history of allergy.

## 2) Intraoperative data

1. Drug use: record the dose and time of anesthesia induction, the total dose and maintenance time during anesthesia maintenance.
2. Hemodynamic data: Record the time point of anesthesia and surgery start, each time point of anesthesia operation, and the end time of trial (data derived from Mediston system)
3. TOF value: record the starting time point of the muscle relaxation monitor and end the monitoring time of the muscle relaxation (the data is exported by the monitor)

### Test flow chart

Pre-test preparation: 1. Ensure the same time; 2. Sign the consent form; 3. record the CRF table; 4. Install NMT accessories; 5. fix the test limb.

Trial start: After sedation and analgesia, start the calibration of NMT in the hands and feet. Select the TOF measurement mode (2Hz, 12s interval)

Inject the muscle relaxant, initiate the measurement;

Administration time, drug name, and dosage were recorded.

The time when the hand reached TOFcnt is 0 and the TOFcnt of the foot at this point

TOFcnt price

After the hand TOFcnt measurement was 0, the interval was maintained as 12s interval (no additional muscle relaxant during the operation)

When the effect gradually subsided, the TOF recovered to the TOF value of the corresponding foot.

The TOFR of the hands and feet reached 90% of the time and the trial ended. Follow-up surgery and anesthesia can be performed as usual according to the specific situation.

## 2.6 Observed indicators

## A) Main evaluation indicators

(1) onset time (OT): the time from cis-atracurium administration to that when TOFC faded to 0;

(2) period of no-twitch response (NTR): the time from cis-atracurium administration to recovery of T1 (TOFC = 1);

(3) spontaneous recovery time (SRT): the time from cis-atracurium administration to when TOFR reached 0.5;

(4) total time (TT): the time from cis-atracurium administration to when TOFR reached 0.9.

## B) Secondary evaluation indicators

Safety measures for neuromuscular monitoring of the foot (hemodynamic data, incidence of adverse reactions)

## 2.7 Data acquisition method

This study is a multicenter clinical study and all data will be recorded in accordance with the GCP to ensure the accuracy, completeness, readability and timeliness of the reported data. In this study, the main observation index TOF was highly correlated with clinical operation, and the value changed dynamically from time to time. In order to accurately record the index change, all data were recorded by electronic data with the Mindray Benevision N12 patient monitor, and the researcher only needed to accurately record the time point of drug administration. Other clinical data will be entered using the Electronic Data Acquisition System (EDC) by the investigator from the original records.

## 2.8 Data quality assurance method

In order to ensure the authenticity and reliability of the data, the data collection is completely automatically recorded by the electronic system (the recording format requires professional tools, and the researcher cannot open it for any intervention), and the investigator only records the administration time point.

The trace target control pump, monitor and monitor of the three research centers can ensure the consistency of drug pump injection and data collection in the three centers. The study has inspectors who regularly visit the three research centers to ensure that the research process does not deviate.

Statisticians do not participate in clinical work, but only according on the data uploaded to the electronic database, according to the research requirements, so as to ensure the objectivity of the statistical results.

**2.9 Data and Statistical Methods**

## 2.9.1 General principles for statistical analysis

The SAS will be used®9.1 or above, descriptive statistics will include mean, standard deviation, median, quartile, minimum, and maximum for continuous variables. For categorical variables, the descriptive statistics will include subjects (N) and percentage.

## 2.9.2 Statistical analysis of the data set

Full Analysis Set (FAS): All subjects who were screened successfully and had at least one valid TOF data.

## 2.9.3, and the distribution of the subjects

The number of subjects involved in screening, successful screening and being included in each analysis set, test completion and early withdrawal (including reasons for withdrawal) were summarized by center.

## 2.9.4 Demographic and baseline data

The demographic data and other baseline characteristics were statistically described according to the center, and for the continuous variables, the mean, median, standard deviation, interquartile, minimum and maximum values were calculated. For the categorical variables, the frequency (N) and the percentage are calculated.

## 2.9.5 TOF values refer to the comparison between digits

The differences between the onset and recovery digits will be analyzed by:

Draw the mean time (± Std) -TOF line plot

Draw the Blant-Altman scatter plot of the difference between the digits during the onset and recovery period, supplemented by the consistency boundaries,

The mean value, standard deviation, and consistency limits of the representative TOF values during the onset and recovery period are summarized.

## 2.9.6, Safety analysis

Summarize the frequency and frequency of adverse events according to the center.

# 3. Matters needing attention

## Factors responsible for muscle relaxation monitoring

1) The influence of the human-machine connection interface

2) The influence of the timing of the control value calibration

3) The influence of the central body temperature and the temperature of the tested site. The Suzuki T[22] study also shows that the skin temperature of the examined site is maintained above 32℃, excluding low temperature on T1 height

influence of.

1. The interaction between the way of each stimulus response
2. Older adults> 65 years

Aging leads to a gradual decline in organ function, reducing the physiological reserves of cardiovascular, nervous, respiratory and liver and kidney systems. Organ decline in the elderly may lead to changes in the pharmacokinetics and pharmacodynamics of anesthetic drugs, prolonging the duration of their action.

1. Halide-inhalation anesthetics

Sevoflurane has a synergistic effect on the relaxation of cis-atracurium and rocuronium [23,24]. ⑴ inhalation anesthetics can reduce the release of acetylcholine by acting on the anterior membrane of the neuromuscular joint and inhibiting the acetylcholine receptor of the neuromuscular joint.⑵ At the same time, by acting on the posterior membrane and muscle membrane of the neuromuscular joint, by changing the Na + -K + ion channel, increasing the threshold of the endplate potential and reducing the sensitivity of the posterior membrane depolarization, thus raising the threshold [26] of the action potential generation by the endplate potential.

1. Different gender effects

Gender leads to differences in the endocrine system, causing different muscle ratios between men and women may affect the incidence of muscle relaxation residual.

Heier [27] et al have shown that there are gender differences in the TOF ratio and the clinical indicators of muscle relaxation recovery. However, due to the small sample size, further studies are needed to confirm the relationship between gender and muscle remnant in the future.

## 4. reference documentation

[1] Gehr LC, Sessler CN.Neuromuscular blockade in the intensive care unit.Semin Respir Crit Care Med.2001;22 (2) :17588.

[2] Rodiera J, Serradell A, AlvarezGomez JA, et al.The cuff method: a pilot study of a new method of monitoring neuromuscular function.Acta Anaesthesiol Scand.2005 Nov;49 (10) :15528.

[3]FUCHS-BUDER T, EIKERMANN M.Residual neuromuscular blockades.Clinical consequences, frequency and avoidance strategies［J］．Anaesthesist, 2006, 55(1):7-16．

[4]MCCAUL C, TOBIN E, BOYLAN JF, et al．Atracurium is associated with postoperative residual curarization［J］．Br J Anaesth, 2002, 89(5):766-769．

[5] MURPHY GS, SZOKOL JW, MARYMONT JH, et al．Intraoperative acceleromyographic monitoring reduces the risk of residual neuromuscular blockade and adverse respiratory events in the postancesthesia care unit［J］． Anesthesiology, 2008, 109(3):389-398．

[6]MURPHY GS, Brull SJ．Residual neuromuscular block： lessons unlearned．Part I： definitions, incidence, and adverse physiologic effects of residual neuromuscular block．Anesth Analg, 2010, 111(1):120-128．

[7]Baillard C, Clec＇h C, Catineau J, et al.Postoperative residual neuromuscular block:a survey of

Management.Br J Anaesth, 2005, 95(5):622-626.

[8] Brief review: Neuromuscular monitoring:an update for the clinician .

[9] Donati F, Plaud B, Meistelman C.A method to measure elicited contraction of laryngeal adductor muscles during anesthesia.Anesthesiology 1991; 74: 827–32.

[10] Churchill-Davidson HC, Christie TH.The diagnosis of neuromuscular block in man.Br J Anaesth 1959; 31: 290–301.

[11] Kirkegaard-Nielsen H, Helbo-Hansen HS, Lindholm P, Pedersen HS, Severinsen IK, Schmidt MB.New equipment for neuromuscular transmission monitoring: a comparison of the TOF-Guard with the Myograph 2000. J Clin Monit Comput 1998; 14: 19–27.

[12] Lina Huang, comparison of adductor pollicis and flexor pollicis for muscle monitoring [J]. The Journal of Clinical Anesthesiology, 2008,

[13] Han Jianxin. The guiding role of muscle relaxation monitoring for vickubromine in clinical anesthesia application [J]. Gansu Medicine, 2008.

[14] TOSHIMITSU KITAJIMA, KEnCHI ISHII, et al .Differential effects of vecuronium on the thumb and the big.Toe muscles evaluated by acceleration measurement.J Anesth (1994) 8:143-145

[15] Aaron F .Kopman et al.Relationship of the train-of-four fade ratio to clinical signs and symptoms of residual paralysis in awake volunteers.[J].Anesthesiology, 1997, 86:765-71.

[16] HEGGERIV, HARBISHETTAR A, DEKA A, et al． Intubating Conditions of two Different Doses of Rocuronium At 60 Seconds;by Clinical Assessment; and with TOF response of Adductor Pollicis Muscle［J］． J Clin Diagn Res, 2015, 9(9):UC24 －28．

[17]Heier T, Hetland S.A comparison of train-of-four monitoring: mechanomyography at the thumb vs acceleromyography at the big toe.Acta Anaesthesiol Scand.1999;43:550–5.

[18] Kitajima T, Ishii K, Kobayashi T, Ogata H.Differential effects ofvecuronium on the thumb and the big toe muscles evaluated by acceleration measurement.J Anesth.1994;8:143–5.

[19]Kern SE, Johnson JO, Orr JA, Westenskow DR.Clinical analysis of the flexor hallucis brevis as an alternative site for monitoring neuromuscular block from mivacurium.J Clin Anesth.1997;9:383–7.

[20]Saitoh Y, Koitabashi Y, Makita K, Tanaka H, Amaha K.Train-of-four and double burst stimulation fade at the great toe and thumb.Can J Anaesth = Journal Canadien D’anesthe´sie.

1997;44:390–5.

[21] Saitoh Y, Fujii Y, Takahashi K, Makita K, Tanaka H, Amaha K.Recovery of post-tetanic count and train-of-four responses at the great toe and thumb.Anaesthesia.1998;53:244–8.

[22] Suzuki T, Kitajima O, Watanabe A, et al.Duration of vecuronium-induced neuromuscular block can be predicted by change of skin temperature over the thenar muscles.J Anesth.2004;18 (3) :1726.

[23] Lowry DW, Mirakhur RK, McCarthy GJ, et al.Neuromuscular effects of rocuronium during sevoflurane, isoflurane, and intravenous anethesia [J].Anesth A nalg, 1998, 87(4):936.

[24] Wulf H, Kahl M, Ledowski T.Augmentation of the neuromuscular blocking effects of cisatracurium during desflurane, sevoflurane, isoflurane or total i.v.anaesthesia [J].Br J Anaesth, 1998, 80(3):308.

[25] Liu Bing, Wen Chao, An Gang. Effect of sevoflurane on the muscle relaxone effect of rocuronium [J]. Journal of Clinical Anesthesiology, 2009,25 (9): 758.

[26] Li Chuanxiang, Yao Shanglong, Nie Hui, et al. Effect of sevoflurane on the effects of nondepolarized muscle relaxants and muscle-type acetylcholine receptors [J]. Chinese Pharmacology Bulletin, 2005,21(9):1081.Computer Methods & Programs in Biomedicine, 2011,104 (2): 154-60

[27]Heier T, Feiner J R, Wright P M C, et al.Sex-related differences in the relationship between acceleromyographic adductor pollicis train-of-four ratio and clinical manifestations of residual

neuromuscular block: a study in healthy volunteers during near steady-state infusion of mivacurium[J].British journal of anaesthesia, 2012, 108(3): 444-451.
