# Supplementary material for: The feasibility of the posterior tibial nerve-flexor hallucis brevis pathway applied in neuromuscular monitoring: a multicentric, controlled, and prospective clinical trial
Source: PeerJ. 2024 Mar 26;12:e17154. doi: 10.7717/peerj.17154 (PMC10979752; doi:10.7717/peerj.17154)
Supplement: Supplemental Information 4 [file peerj-12-17154-s004.docx]

**
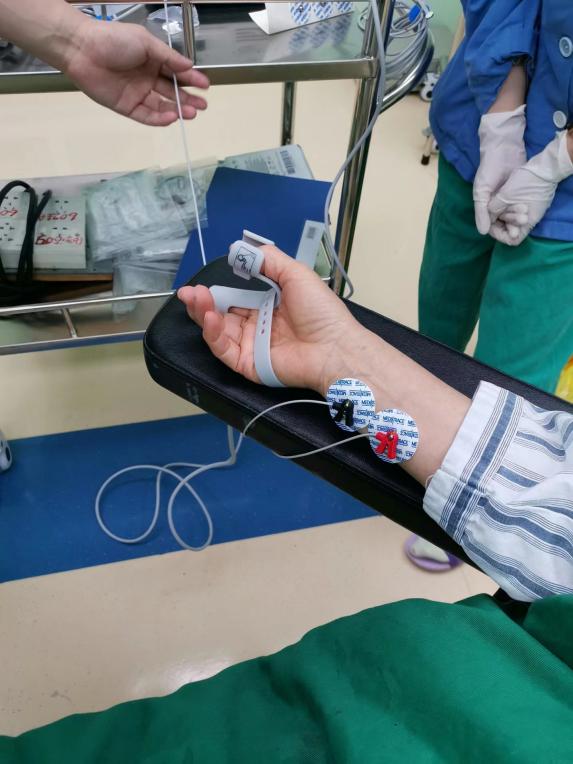
**

Figure 1 Monitoring diagrams of the ulnar nerve-adductor pollicis pathway.


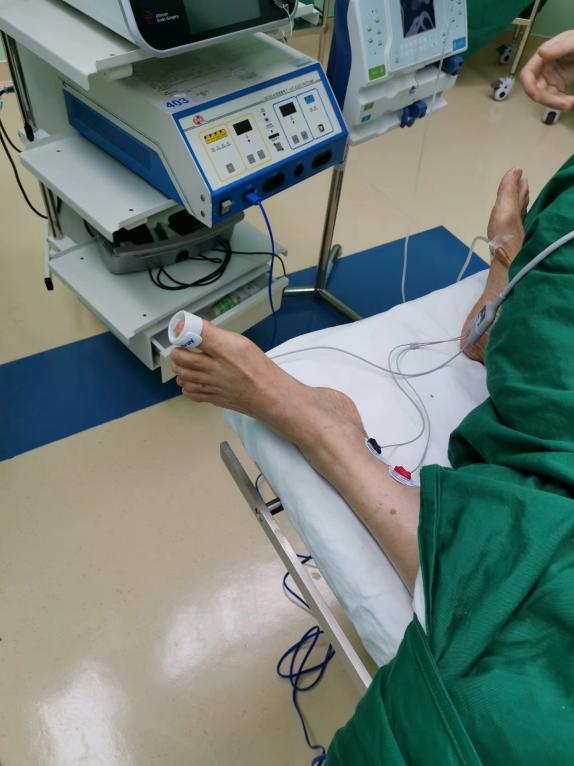


Figure 2 Monitoring diagrams of the posterior tibial nerve-flexor hallucis brevis pathway.
