# Supplementary material for: The feasibility of the posterior tibial nerve-flexor hallucis brevis pathway applied in neuromuscular monitoring: a multicentric, controlled, and prospective clinical trial
Source: PeerJ. 2024 Mar 26;12:e17154. doi: 10.7717/peerj.17154 (PMC10979752; doi:10.7717/peerj.17154)
Supplement: Supplemental Information 1 [file peerj-12-17154-s001.zip › Raw data/Figure 3/figure 3.doc]

**Figure 2** The Bland-Altman plots of monitoring results between thumb and toe, including OT (A), NTR (B), SRT (C), and TT (D).

**Figure 3** The percentages of the counts of +/- difference of monitoring results, including OT (A), NTR (B), SRT (C), and TT (D). Positive difference means the period of thumb > toe while negative difference means thumb ≤ toe. All the percentages between thumb and toe reached significant (all P<0.001).
