# Supplementary material for: The feasibility of the posterior tibial nerve-flexor hallucis brevis pathway applied in neuromuscular monitoring: a multicentric, controlled, and prospective clinical trial
Source: PeerJ. 2024 Mar 26;12:e17154. doi: 10.7717/peerj.17154 (PMC10979752; doi:10.7717/peerj.17154)
Supplement: Supplemental Information 1 [file peerj-12-17154-s001.zip › Raw data/table 1/table1.doc]

**Table 1 Patient’s demographic characteristics**

| Parameters | Mean±SD, N (%) |
| --- | --- |
| Age, year | 43.70±15.43 |
| Sex |  |
| Male | 66 (42.86%) |
| Female | 88 (57.14%) |
| Height, cm | 163.58±7.76 |
| Weight, kg | 61.48±10.67 |
| BMI, kg/m2 | 22.92±3.32 |
| ASA |  |
| I | 109 (70.78%) |
| Ⅱ | 45(29.22%) |
| Source |  |
| Center S | 73 (50.65%) |
| Center G | 38 (22.73%) |
| Center J | 43 (26.62%) |
| Surgical type |  |
| General surgery | 69 (44.80%) |
| Otalaryngology | 69 (44.80%) |
| Gynecology | 12 (7.79%) |
| Orthopedics | 4 (2.59%) |
